# Is lower-dose sugammadex a cost-saving strategy for reversal of deep neuromuscular block? Facts and fiction

**DOI:** 10.1186/s12871-018-0605-6

**Published:** 2018-11-06

**Authors:** Hans D de Boer, Ricardo V Carlos, Sorin J Brull

**Affiliations:** 1 Department of Anesthesiology and Pain Medicine, Martini General Hospital Groningen, Groningen, the Netherlands; 2 Department of Anesthesiology, Child Institute, Hospital das Clinicas, Sao Paulo University Medical School, Sao Paulo, Brazil; 3 Department of Anesthesiology and Perioperative Medicine, Mayo Clinic College of Medicine, Jacksonville, Florida USA

**Keywords:** Sugammadex, Reversal of neuromuscular block, Reversal drug under-dosing, Residual and recurring neuromuscular block, Neuromuscular monitoring

## Abstract

**Background:**

Sugammadex, a γ-cyclodextrin derivative, belongs to a new class of selective relaxant binding agents. Sugammadex was approved 10-years ago by the European medicines agency and today is used in clinical anesthesia and emergency medicine globally. In this review, indications for neuromuscular block, the challenge of neuromuscular monitoring and the practice of under-dosing of sugammadex as a potential cost-saving strategy are discussed.

**Main body:**

Reversal of neuromuscular block is important to accelerate the spontaneous recovery of neuromuscular function. Sugammadex is able to reverse a rocuronium- or vecuronium-induced neuromuscular block rapidly and efficiently from every depth of neuromuscular block. However, since sugammadex was introduced in clinical anesthesia, several studies have reported administration of a lower-than-recommended dose of sugammadex. The decision to under-dose sugammadex is often motivated by cost reduction concerns, as the price of sugammadex is much higher than that of neostigmine outside the United States. However, under-dosing of sugammadex leads to an increased risk of recurrence of neuromuscular block after an initial successful (but transient) reversal. Similarly, when not using objective neuromuscular monitoring, under-dosing of sugammadex may result in residual neuromuscular block in the postoperative care unit, with its attendant negative pulmonary outcomes. Therefore, an appropriate dose of sugammadex, based on objective determination of the depth of neuromuscular block, should be administered to avoid residual or recurrent neuromuscular block and attendant postoperative complications. Whether the reduction in perioperative recovery time of the patient can be translated into additional procedural cases performed, faster operative turnover times, or improved organizational resource utilization, has yet to be determined in actual clinical practice that includes verification of neuromuscular recovery prior to tracheal extubation.

**Conclusions:**

The current review addresses the indications for neuromuscular block, the challenge of neuromuscular monitoring, the practice of under-dosing of sugammadex as a potential cost-saving strategy in reversal of deep neuromuscular block, the economics of sugammadex administration and the potential healthcare cost-saving strategies.

## Background

A study by Aouad et al. in BMC Anesthesiology [1] investigated the efficacy of the combination of half-dose sugammadex plus neostigmine to reverse a rocuronium-induced deep neuromuscular block. The authors discussed the potential for lowering the total costs of the neuromuscular block reversal strategy due to the relatively high cost of sugammadex compared with that of neostigmine. This study showed interesting results, and the authors are to be commended for their ingenuity in attempting to lower the cost of anesthesia care. However, their methods also have raised some counterarguments that we feel should be addressed.

Over 75 years ago, Harold Griffith and Enid Johnson published their famous paper on the use of curare in general anesthesia [2]. The introduction of curare allowed adequate muscle relaxation at a lighter, and therefore better-tolerated, depth of general anesthesia. However, in 1954 Beecher and Todd [3] reported a study of the mortality associated with anesthesia and surgery, in which they found that mortality rates were six times higher when neuromuscular blocking drugs were used, and that 63% of the deaths were caused by respiratory failure. Since then, many publications have reported the risks of using neuromuscular blocking drug and the concomitant high incidence of residual neuromuscular block [4–8]. Therefore, antagonism of neuromuscular blocking agents is essential when using these drugs, and the appropriate drug and dose should be based on assessment of objective (quantitative) neuromuscular monitoring.

Following approval of sugammadex for clinical use, many publications investigated the adequate sugammadex dosing, including the combination of sugammadex and cholinesterase inhibitors as strategies to decrease the cost of sugammadex antagonism. Aouad et al. reported a study in which either sugammadex alone or a combination of sugammadex plus neostigmine was used to reverse a rocuronium-induced deep neuromuscular block. The current review addresses the indications for reversal of neuromuscular block, the challenge of neuromuscular monitoring, the practice of under-dosing of sugammadex as a potential cost-saving strategy in reversal of deep neuromuscular block, and the healthcare economics of sugammadex as cost-saving strategies.

## Review

### Indications for reversal of neuromuscular block

There are few medications that are still in use today, more than 80 years after their first introduction into clinical use. Neostigmine is one of those rare medications: it was first synthesized in 1931, and was patented by Aeschlimann in 1933 [9]. Since that time, it has been used in the treatment of a variety of conditions such as myasthenia gravis, Ogilvie’s syndrome, and as an antidote for snakebite venom. The most common clinical use is for inhibiting acetylcholinesterases, thereby antagonizing the effects of nondepolarizing neuromuscular blocking agents (NMBAs).

Although in clinical use for many decades, neostigmine has well-known side effects (such as bradycardia, nausea, vomiting, abdominal cramping, diarrhea) and limitations. To understand the latter, it is important to remember neostigmine’s mode of action: it inhibits acetylcholinesterases, so that acetylcholine that is released from the presynaptic nerve terminal is no longer broken down. The maximal concentration of acetylcholine that can be achieved at the neuromuscular junction in order to compete with NMBAs for receptor binding at the muscle terminal is therefore limited by the amount that is released from presynaptic stores. Once acetylcholinesterases are maximally inhibited by the administered neostigmine, any additional neostigmine will be unable to further increase the amount of acetylcholine at the neuromuscular junction. This results in several important limitations: at deep degrees of neuromuscular block (defined as no response to train-of-four [TOF] stimulation, when the NMBA concentration at the neuromuscular junction is very high), neostigmine is ineffective in producing adequate reversal of block. [10–12] A suggested and updated set of definitions of the depth of neuromuscular block (based on both subjective and objective criteria) was recently published (Table 1). [13]

**Table 1:** Definitions of Depth of Neuromuscular Block Based on Subjective and Measured (Objective) Criteria

Depth of block	Posttetanic	Train-of-Four	Subjective	Measured
	Count	Count	Train-of-Four ratio	Train-of-Four ratio
Intense (profound) block	0	0	0	0
Deep block	≥ 1	0	0	0
Moderate block	NA	1 to 3	0	0
light (shallow) block	NA	4	Fade present	0.1–0.4
Minimal block (near recovery)	NA	4	No fade	> 0.4 but < 0.90
Full recovery (normal function)	NA	4	No fade	≥ 0.90–1.0

*NA* not applicable

Adapted from: Brull SJ, Kopman AF. Current status of neuromuscular reversal and monitoring: challenges and opportunities. Anesthesiology 2017; 126:173–90 [13]

Conversely, once recovery of function is nearly complete (or at full recovery), neostigmine may impair genioglossus muscle activity, leading to upper airway collapse [14, 15]. However, in a recently published clinical trial it was shown that neostigmine (40 mcg/kg) given to patients who had recovered to a TOF-ratio >  0.9 did not result in clinically relevant neostigmine-induced muscle weakness. [16] In contrast to previous findings, this study suggested that postoperative patients may not exhibit overt signs and symptoms of neuromuscular weakness after receiving a single, moderate dose of neostigmine at near-full recovery. Because of neostigmine’s side effects and limitations, new pharmacologic antagonists have been developed. Sugammadex, a cyclodextrin compound, irreversibly binds to aminosteroid NMBAs in the plasma, but has no activity against benzylisoquinolinium compounds (atracurium, cisatracurium) or succinylcholine [17–19]. Unlike neostigmine, which indirectly reverses nondepolarizing block by increasing the concentration of acetylcholine at the neuromuscular junction, sugammadex directly and effectively inactivates steroidal nondepolarizing NMBAs and their neuromuscular blocking activity.

### The challenge of neuromuscular monitoring

The literature is replete with studies, reports, review articles, letters and editorials documenting the inadequacy of subjective (tactile and visual) evaluation of neuromuscular function, particularly once the train-of-four (TOF) ratio is > 0.40. Similarly, clinical tests (such as the ubiquitous 5-s head lift) have a very low positive predictive value and specificity [20, 21]. Despite these limitations, most clinicians continue to rely on subjective evaluation and/or clinical tests in making decisions about the adequacy of neuromuscular function prior to tracheal extubation [22]. The reasons for the continued (and erroneous) use of these criteria are varied, and reflect mostly the common misunderstanding of the limitations of qualitative (subjective) assessments. The problem is compounded by the unavailability of an easy to use, reliable (objective) monitor. The acceleromyography (AMG)-based TOF-Watch (Organon, Dublin, Ireland), the most commonly used monitor, is no longer available. Other stand-alone AMG-based monitors exist (TOFscan, IDMed, France; Stimpod, Xavant Technology, Ltd., South Africa), as well as EMG-based monitors (TetraGraph, Senzime, Sweden). Additionally, there are workstation-integrated modules based on kinemyography (KMG) and electromyography (EMG) (GE Healthcare, WI, USA) [13].

### Use of low-dose sugammadex

Since sugammadex was introduced in clinical anesthesia, several studies have reported administration of a lower-than-recommended dose of sugammadex. The decision to under-dose sugammadex is often motivated by cost reduction strategies, as the price of sugammadex is much higher than that of neostigmine outside the United States. However, using lower-than-recommended doses (under-dosing) leads to an increased risk of recurrence of neuromuscular block after initial successful (but transient) reversal. Similarly, when not using objective neuromuscular monitoring, under-dosing of sugammadex may result in residual neuromuscular block in the postoperative care unit, with its attendant negative pulmonary outcome [23].

In an early dose-finding study by Eleveld et al., a case was described in which a temporary decrease of the TOF ratio was observed in a healthy patient after reversal of a rocuronium-induced NMB with 0.5 mg/kg sugammadex. The initial peak TOF ratio during recovery was 0.7, but then it decreased to 0.30, and then gradually returned to 0.9. The authors hypothesized that the initial dose of sugammadex effectively bound the rocuronium molecules, leading to the initial recovery of the TOF to 0.7; however, redistribution of free (unbound) rocuronium from the peripheral compartments back into the plasma led to the subsequent recurrence of neuromuscular block. [24] While it is possible that that the transient decrease in TOF ratio was the result of the first twitch (T_1_) increasing faster than the fourth twitch (T_4_), the individual T_1_ and T_4_ amplitudes were not reported, and this latter mechanism cannot be fully excluded.

Similarly, inadequate reversal of a rocuronium-induced deep NMB was described in two healthy patients given suboptimal doses of sugammadex (0.5 mg/kg) [25]. Duvaldestin et al., investigated reversal of a deep NMB with low-dose sugammadex (0.5 and 1.0 mg/kg), and found that five patients experienced recurrence of NMB. [26] Both speed of reversal and effectiveness of sugammadex are dose-dependent; a single 1-mg/kg dose of sugammadex (*n* = 15 patients) administered at a deep rocuronium block (PTC = 1) required significantly longer time for reversal than a dose of 4.0 mg/kg (*n* = 60) [27]. Interestingly, seven patients also exhibited residual (*n* = 3) and recurrent (*n* = 4) neuromuscular block in the group of patients who received 1.0 mg/kg sugammadex. No patients who received the recommended dose of 4.0 mg/kg sugammadex had residual or recurrent neuromuscular block. [27] The findings also underscore the importance of objective (quantitative) monitoring in order to diagnose incomplete recovery and avoid postoperative sequelae [22]. A similar pattern was observed with vecuronium-induced block: a longer reversal time and a higher incidence of residual and/or recurring block were noted in patients who received smaller (0.5 and 1.0 mg/kg) doses of sugammadex. [23] However, the literature of this phenomenon in a vecuronium-induced NMB is sparse.

Another study found that when any TOF fade is observed, a low dose of sugammadex 0.5–1.0 mg/kg will result in a more reliable and faster recovery to a TOF 0.9 than clinically used doses of neostigmine. [28] However, when using these low doses of sugammadex there is an increased risk of residual NMB or recurrence of NMB. Moreover, administration of sugammadex doses based on empiric or subjective assessment of the depth of block is not protective: an average dose of 2.7 mg/kg of sugammadex administered to patients managed intraoperatively without objective neuromuscular monitoring resulted in an incidence of TOF ratio < 0.9 at the time of tracheal extubation as high as 9.4%. [29] The studies that continue to report residual or recurrent neuromuscular block, however, do not suggest pharmacologic limitations of sugammadex; the occurrence of residual or recurrent block, and/or their attendant complications, is due to inadequate assessment of sufficient neuromuscular recovery at the time of tracheal extubation. If tracheal extubation were performed at the time of recovery of TOF ratio to 1.0, there would be no complications ascribed to residual block.

Therefore, a sufficiently large dose of sugammadex (ideally, one based on objective determination of the depth of neuromuscular block) should be administered, and tracheal extubation should be performed only when adequate recovery is documented by objective means in order to avoid residual or recurrent neuromuscular block and attendant postoperative complications.

### Health economics of sugammadex and cost-saving strategies

Under-dosing of sugammadex is often based on cost reduction strategies, as the price of sugammadex compared with neostigmine is high in countries other than the United States [27]. However, no health economic studies investigated the cost savings of a low dose compared with the recommended dose of sugammadex. The cost effectiveness of sugammadex is difficult to determine, as many various confounders exist. A systematic review assessed the cost effectiveness of sugammadex vs. neostigmine for reversal of either moderate or profound NMB induced by rocuronium or vecuronium [30]. This assessment was based on early clinical trials and showed that sugammadex use would be cost-effective if it reduced the more expensive operating room time, but not if it only reduced the recovery room time. [30] Other investigators have shown that residual neuromuscular block was associated with a longer PACU length of stay, and additionally, the presence of residual weakness also significantly increased “the chances of patients having to wait to enter the PACU” – presumably by delaying the transfer of patients from the operating room. [31]

Other analyses explored the potential impact of sugammadex use on operating room efficiency and cost. [32] The discrete event simulation model developed found that when full neuromuscular recovery was verified prior to tracheal extubation, the use of sugammadex (rather than neostigmine) avoided 2.4 procedural cancellations “due to OR time over-run and 33.5 h of paid staff overtime, while saving an average of 62 min per OR day.” [32] Whether the reduction in perioperative recovery time of the patient can be translated into additional procedural cases, faster turnover times, or improved organizational resource utilization, has to be determined in actual clinical practice that includes verification of neuromuscular recovery (by objective monitoring) prior to tracheal extubation. [32]

While the technique of “dose-splitting” (using the same single vial of medication for multiple patients) may indeed be cost-effective in some cases, such practice is not recommended and is purposefully excluded from discussion here.

Multiple unanswered questions remain on whether sugammadex is cost effective in reversing NMB. It is to be expected that underdosing of sugammadex may result in prolonged recovery times and more frequent complications due to RNMB and recurrence of NMB, which also may increase concomitant healthcare cost. More prospective studies are needed to answer these important economic and patient safety questions.

## Conclusions

The current review addresses the indications for reversal of neuromuscular block, the challenge of neuromuscular monitoring, the under-dosing of sugammadex as a potential cost-saving strategy in reversal of deep neuromuscular block and the healthcare economics of sugammadex and cost-saving strategies. Reversal of neuromuscular block is important to prevent residual neuromuscular paralysis and concomitant morbidity and mortality. Despite many publications, most clinicians continue to rely on subjective evaluation and/or clinical tests in making decisions about the adequacy of neuromuscular function prior to tracheal extubation. Objective neuromuscular monitoring should be used when neuromuscular blocking drugs are used to determine the level of neuromuscular block and whether there is an indication for reversal of neuromuscular block. Under-dosing of sugammadex leads to an increased risk of recurrence of neuromuscular block after initial successful (but transient) reversal. Therefore, an appropriate large dose of sugammadex based on objective determination of the depth of neuromuscular block should be administered to avoid residual or recurrent neuromuscular block and attendant postoperative complications. Underdosing of sugammadex is often based on cost reduction strategies, but to-date, no health economic studies investigated the cost savings of a low dose compared with the recommended dose of sugammadex. The cost effectiveness of sugammadex is difficult to determine, as many various confounders exist. Whether the reduction in perioperative recovery time of the patient can be translated into additional procedural cases, faster turnover times, or improved organizational resource utilization, has to be determined in actual clinical practice that includes verification of neuromuscular recovery prior to tracheal extubation.

## Abbreviations

NMB: neuromuscular block
NMBA: neuromuscular blocking agent
OR: operation room
PACU: post anesthesia care unit
RNMB: residual neuromuscular
TOF: train-of-four
TOFR: train-of-four ratio
